# The Fallacy of Small Numbers

**Published:** 1901-06-29

**Authors:** 


### THE FALLACY OF SMALL NUMBERS.

Conclusions based upon statistics are notoriously open to many fallacies, and the smaller the numbers on which hey are based the more ridiculous are the conclusions which it is possible to deduce from them. In the *Polyclinic* of this month there is a short article the aim of which is, apparently, to poke fun at some of the statistics based on small numbers which are published from time to time about insanity. During the year 1900, we are told, 226 patients were admitted as lunatics into Bethlem Hospital, and from a table which records "religious persuasion" it is possible to obtain a series of facts, "which, let it be noted, however imposing they may seem if cited tn an argument, really prove nothing." After arranging the cases according to their religious persuasions and ascertaining the results of treatment in the several groups, it is shown that if we were to overlook the fact that the numbers are so small as to make the results entirely misleading, it might be argued from the facts presented "that members of the Established Church, when they become mentally deranged are difficult of cure, and Wesleyan yet more so; whilst Baptists, Roman Catholics, Jews, and those of lax creeds fare much better," all of which we may safely say is absurd.

